# “Taking away the chaos”: a health needs assessment for people who inject drugs in public places in Glasgow, Scotland

**DOI:** 10.1186/s12889-018-5718-9

**Published:** 2018-07-04

**Authors:** Emily J. Tweed, Mark Rodgers, Saket Priyadarshi, Emilia Crighton

**Affiliations:** 1 Directorate of Public Health, NHS Greater Glasgow and Clyde, West House, Gartnavel Royal Hospital, 1055 Great Western Road, Glasgow, G11 0SX UK; 2 MRC/CSO Social and Public Health Sciences Unit, University of Glasgow, 200 Renfield Street, Glasgow, G2 3QB UK; 3 NHS Greater Glasgow and Clyde, JB Russell House, Gartnavel Royal Hospital, 1055 Great Western Road, Glasgow, G11 0SX UK; 4 School of Medicine, Dentistry, and Nursing, University of Glasgow, Glasgow, G12 8QQ UK; 5 NHS Greater Glasgow and Clyde Addictions Services, Festival Business Centre, 150 Brand Street, Glasgow, G51 1DP UK

**Keywords:** Drug use, Public injecting, Health needs assessment

## Abstract

**Background:**

Public injecting of recreational drugs has been documented in a number of cities worldwide and was a key risk factor in a HIV outbreak in Glasgow, Scotland during 2015. We investigated the characteristics and health needs of people involved in this practice and explored stakeholder attitudes to new harm reduction interventions.

**Methods:**

We used a tripartite health needs assessment framework, comprising epidemiological, comparative, and corporate approaches. We undertook an analysis of local and national secondary data sources on drug use; a series of rapid literature reviews; and an engagement exercise with people currently injecting in public places, people in recovery from injecting drug use, and staff from relevant health and social services.

**Results:**

Between 400 and 500 individuals are estimated to regularly inject in public places in Glasgow city centre: most experience a combination of profound social vulnerabilities. Priority health needs comprise addictions care; prevention and treatment of blood-borne viruses; other injecting-related infections and injuries; and overdose and drug-related death. Among people with lived experience and staff from relevant health and social care services, there was widespread – though not unanimous – support for the introduction of safer injecting facilities and heroin-assisted treatment services.

**Conclusions:**

The environment and context in which drug consumption occurs is a key determinant of harm, and is inextricably linked to upstream social factors. Public injecting therefore requires a multifaceted response. Though evidence-based interventions exist, their implementation internationally is variable: understanding the attitudes of key stakeholders provides important insights into local facilitators and barriers. Following this study, Glasgow plans to establish the world’s first co-located safer injecting facility and heroin-assisted treatment service.

**Electronic supplementary material:**

The online version of this article (10.1186/s12889-018-5718-9) contains supplementary material, which is available to authorized users.

## Background

Public injecting is commonly defined as the injecting of recreational drugs in places accessible to the general public, such as alleyways, public toilets, and stairwells [1, 2]. It has been described in a number of cities worldwide, including London, Baltimore, Vancouver, Sydney, and Dublin [3–7], and has been identified as an important factor in an ongoing HIV outbreak among people who inject drugs in Glasgow, Scotland’s largest city [8].

However, there is little up-to-date evidence as to the characteristics or health needs of people who inject drugs in public places, particularly outside North America or Australia.

Some cities affected by public injecting have introduced safer injecting facilities and heroin-assisted treatment as harm reduction measures [9–11]. Safer injecting facilities (SIFs) are hygienic environments where illicit drugs – purchased off the premises – can be consumed under clinical supervision [10, 12]. Heroin-assisted treatment (HAT) refers to the prescription of pharmaceutical heroin (diamorphine) by medical professionals to treat opiate-dependent individuals who do not benefit from existing substitution therapies, such as methadone or buprenorphine [11, 13].

Both of these interventions have been recommended by national and international bodies such as the UK Advisory Council on the Misuse of Drugs and the European Monitoring Centre for Drugs and Drug Addiction [14–16], but implementation to date has been limited. In the UK, for instance, no safer injecting facility has ever operated and HAT is not currently routinely available from addictions services.

Understanding facilitators and barriers to implementation of these interventions is therefore important. However, few studies have examined this area. In particular, attitudes of professional stakeholders from health, social care, and law enforcement have not (to our knowledge) previously been described.

This study aimed to investigate the characteristics and health needs of people who inject drugs in public in Glasgow, Scotland. It also examined factors relevant to the implementation of safer injecting facilities and heroin-assisted treatment, which are not currently provided anywhere in the UK.

## Methods

We used an established tripartite framework for health needs assessment [17], comprising the following strands:

### Epidemiological assessment

We collated and analysed secondary data from a range of local sources, including injecting equipment provision (IEP) outlets, the city council Land and Environmental Services (responsible for clearing drug-related litter) the Scottish Ambulance Service, Community Safety Glasgow, and a drug-related deaths database. Due to space constraints, only results from the first two of these are presented in this article: details of these sources and guidance on their interpretation are provided in Table 1. Results from the other analyses are available from the full report.

**Table 1:** Data sources presented in epidemiological strand of needs assessment

Name	Description	Guidance on interpretation
Injecting Equipment Provision (IEP) clients and transactions	Demographic and clinical details, and transaction history, for individuals utilising IEP outlets in the city centre. Based on data from the Neo database, which covers all IEP services across NHS Greater Glasgow and Clyde.	Data are limited to clients reporting injecting heroin and/or cocaine, to exclude users of performance- and image-enhancing drugs, who have a different epidemiological profile and set of health needs. Pilot analyses demonstrated that injecting of other drugs (such as novel psychoactive substances) among city centre IEP attendees was negligible. A degree of duplication may exist within the Neo system, with some individuals having registered on more than one occasion. Data are therefore presented separately for clients using any of the seven IEP outlets in the city centre during 2015 those with ≥5 transactions during 2015 (denoted ‘repeat clients’; a proxy for unique users) those with ≥50 transactions during 2015 (denoted ‘high-frequency clients’) Since location of use is not currently recorded in Neo, data are also presented separately for clients accessing IEP via the Assertive Outreach team: this is a service set up to address the needs of public injectors and is therefore the best proxy indicator for public injecting.
Drug-related litter	Reports of drug-related litter made to Glasgow City Council’s Land & Environmental Services (LES) department	These data do not include information on volume of litter (e.g. number of needles) or any clean-ups undertaken on private property by individuals or companies. They also rely on reporting by members of the public. These data are therefore presented to indicate the likely geographical distribution of public injecting but will underestimate its extent.

A series of rapid literature reviews were undertaken to identify published research on the health needs of people who inject drugs in public places and to evaluate the evidence for the specific interventions of interest. Details of search strategies and results are available online (Additional file 1).

### Comparative assessment

Information on services provided in other regions and countries for people who inject drugs in public places was gathered during the literature review (Additional file 1). Performance data for local addictions services were collated from a Scotland-wide surveillance study of people who inject drugs [18], the national Drug and Alcohol Treatment Waiting Times and Drug-Related Deaths databases [19, 20], and local data on opioid substitution therapy prescribing.

### Corporate assessment

In order to understand stakeholder perspectives on public injecting, a three-part engagement exercise was undertaken as follows:

- Six semi-structured interviews with people who inject drugs in the city centre, recruited through an existing Assertive Outreach service;
- a focus group with 15 people in recovery from drug use, recruited through a local peer network; and
- an online survey with 33 staff from relevant health services, community services, support organisations, and enforcement agencies.

The engagement exercise focused on three topics: the health needs of people who inject drugs in public places; experiences of current services; and attitudes to potential novel services. Question schedules are available in the online Additional file 2. The interviews and focus group were audio recorded and subsequently transcribed. Results of the consultation were analysed using the framework method with the support of an experienced qualitative researcher.

### Ethics, consent, and permissions

This project was undertaken as part of routine service monitoring and quality improvement, so ethics committee approval was not required [21]. All stakeholders who participated in interviews and focus groups gave verbal informed consent prior to participation. Verbal (rather than written) consent was chosen given the nature of the project as a routine quality improvement initiative, and for practical reasons in the case of focus groups. Participant consent was recorded in the transcripts of the interviews and focus groups. For stakeholders who participated in the online engagement exercise, the survey included an information page and contact details in case of any queries, and participants were asked to select from a series of options as to their consent to participate and their willingness to be directly quoted in any publications.

## Results

### Who is injecting in public?

Data from local services targeting people involved in public injecting suggest that this population is predominantly male, aged between 30 and 50 years, and of Scottish ethnic origin (Table 2). The high rate of homelessness observed locally in this population was corroborated by published research showing that public injecting is closely associated with a combination of social vulnerabilities, including homelessness and housing insecurity, offending, and destitution [1, 4, 22–25]. By applying estimates of the prevalence of public injecting in three UK cities to local IEP data, we estimated that between 400 and 500 individuals may be injecting in public places in Glasgow city centre on a regular basis (Additional file 3).

**Table 2:** Characteristics of people using city centre IEP outlets during 2015 who reported injecting heroin and/or cocaine

	All clients (%)	‘Regular clients’ ≥5 transactions (%)	‘High frequency clients’ ≥50 transactions (%)	Clients receiving IEP via Assertive Outreach^a^ (%)
Agegroup^b^				
< 20 years	10 (0.3)	2 (0.2)	0 (0.0)	0 (0.0)
20–29 years	318 (9.6)	87 (8.5)	21 (14.9)	43 (14.5)
30–39 years	1423 (42.9)	444 (43.3)	54 (38.3)	132 (44.4)
40–49 years	1297 (39.1)	394 (38.4)	54 (38.3)	104 (35.0)
≥ 50 years	272 (8.2)	98 (9.6)	12 (8.5)	18 (6.1)
Gender				
Male	2702 (81.4)	850 (82.9)	118 (83.7)	244 (82.2)
Female	618 (18.6)	175 (17.1)	23 (16.3)	53 (17.8)
Ethnicity				
Scottish	3075 (92.6)	956 (93.3)	133 (94.3)	277 (93.9)
Other white ethnic group^c^	181 (5.5)	48 (4.7)	7 (5.0)	14 (4.7)
Other ethnic group^d^	41 (1.2)	14 (1.4)	1 (0.7)	1 (0.3)
Unknown	23 (0.7)	7 (0.7)	0 (0.0)	3 (1.0)
Last recorded housing status^e^				
Owner or renting	2448 (73.7)	697 (68.0)	79 (56.0)	121 (40.7)
Homeless	755 (22.7)	276 (26.9)	46 (32.6)	127 (42.8)
Roofless	114 (3.4)	52 (5.1)	16 (11.3)	46 (15.5)
Unknown	3 (0.1)	0 (0.0)	0 (0.0)	3 (1.0)
Primary drugs of injection^f^				
Heroin only	2682 (86.6)	826 (80.6)	113 (80.1)	237 (79.8)
Both heroin & cocaine	228 (6.9)	127 (12.4)	24 (17.0)	36 (12.1)
Cocaine only	187 (5.6)	34 (3.3)	1 (0.7)	3 (1.0)
Incomplete/unknown^g^	223 (6.7)	38 (3.7)	3 (2.1)	24 (8.1)
Last recorded treatment status^h^				
In structured treatment	538 (16.2)	219 (22.9)	44 (32.4)	109 (36.7)
Not in structured treatment	716 (21.6)	308 (32.3)	50 (36.8)	133 (44.8)
Prefer not to say	1670 (50.3)	431 (45.0)	42 (30.9)	50 (16.8)
No answer	396 (11.9)	67 (7.0)	5 (3.7)	5 (1.7)
Total	3320	1025	141	297

^a^Note that this is a subset of the total number of clients (‘All clients’) and will include individuals from the ‘regular’ and ‘high frequency’ client groups. It is shown separately to highlight the characteristics of this subset of IEP clients most likely to be involved in public injecting

^b^As recorded at most recent transaction

^c^Census codes 1B-1Z

^d^ Census codes 2A, 3F-3Z, 4D-4Y, 5C-5Y, 6A-6Z

^e^As recorded at most recent transaction. Homeless defined as living in temporary or unstable accommodation; roofless defined as rough sleeping

^f^Individuals can have more than one primary drug of injection

^g^Primary drug of injection is not a mandatory field so may be left incomplete, even if information on drugs used by that client is available from other fields

^h^As recorded at most recent transaction. Structured treatment defined as tier 3 or 4 services (see reference [41])

### Why – and where – is public injecting taking place?

Engagement with people currently injecting drugs and those in recovery suggested that public injecting represents a complex trade-off between wanting to inject immediately after acquiring drugs – because of withdrawal symptoms and a fear of arrest or robbery – and feelings of shame, and a wish not to be seen by the general public. Lack of private space in which to inject was also a key factor. These themes are consistent with previous qualitative research among public injectors [2, 3, 26–29].

*“I had to go down below a bridge to inject with other using addicts, as a result of if I get caught doing it in the [homeless] hostel, I would have been papped out.”*

*Focus group participant (in recovery).*

**Fig. 1:**
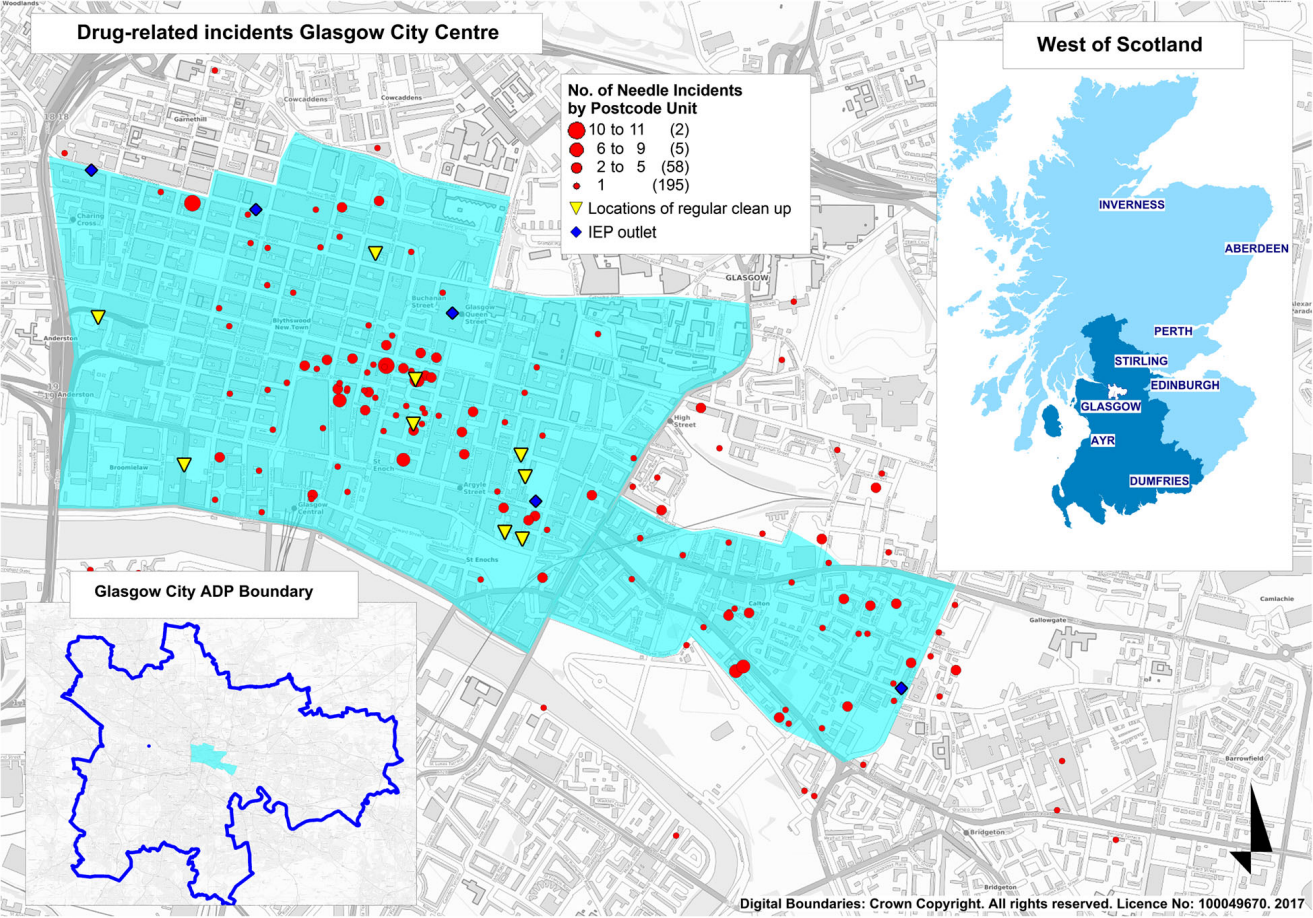
Number of drug-related litter incidents in the city centre and surrounding areas during 2015

Mapping reports of drug-related litter and incidents of drug misuse indicated that public injecting in Glasgow is concentrated in the south-east area of the city centre (Fig. 1). These data and our stakeholder engagement identified a range of public injecting sites, including alleyways, car parks, stairwells, public toilets, and wastelands. The choice of location was largely dictated by the geography of local drug markets and the desire for privacy and shelter.

*“There aren’t really any places to go. As you say, it’s like public toilets or things like that you’re needing to go to, and obviously you’re taking the chance of getting caught.”*

*Interviewee (currently injecting drugs).*

### What are the health needs of people who inject in public places?

People with lived experience of injecting drug use reported that health was a low priority during periods of active drug use, despite a high level of need. Participants highlighted a number of reasons for this, including the demands of their addiction, adverse social circumstances, fatalistic attitudes towards health, and fears of potential repercussions or stigma when seeking help.

*“[When asked about barriers to better health] Just this life I’ve got just now. Terrible. Being homeless and all that, running about the city centre, shoplifting, begging, just doing anything to make money.”*

*Interviewee (currently injecting drugs).*

“*To be honest, I’m just ravaged wi’ addiction and when I’m ravaged I kind of cannae take care of myself.”*

*Interviewee (currently injecting drugs).*

Four key health needs were identified from our synthesis of local data and the published literature, as follows.

#### Addictions care

The literature review found that public injecting is associated with a higher intensity of addiction, as indicated by injecting frequency, number of bodily injecting sites, poly-drug use, and validated measures of dependency [10–12, 18]. Locally, 37% of individuals known to the Assertive Outreach team reported current structured addictions treatment, suggesting a sizeable population for whom existing treatment options may be failing to reduce street drug use (Table 2). Stakeholder attitudes to local addictions care were generally very favourable, but some staff identified challenges in providing sufficiently intensive or flexible care for this population and a need for a greater focus on harm reduction.

#### Blood-borne virus risk

The literature review identified an association between public injecting and: sharing injecting equipment; injecting in larger groups; and inappropriate disposal of needles and syringes [3, 4, 7, 22–25, 30, 31]. A smaller number of studies have suggested that public injecting is also associated with higher prevalence of blood-borne viruses [23, 32]. Local data show that people known to city centre IEP outlets tend to take fewer needles at each transaction – a risk factor for sharing – and have much lower rates of return of used needles for safe disposal, compared to other IEP outlets in the area (Table 3).

**Table 3:** Transactions at city centre IEP outlets during 2015 made by clients who reported injecting heroin and/or cocaine

	All clients	‘Regular clients’ (≥5 transactions)	‘High frequency clients’ (≥50 transactions)	Clients of Assertive Outreach team
Total number of transactions	31,298	27,526	13,735	2325
Equipment provided				
All needles	262,480	189,752	69,365	12,778
Longer needles for deep vein injection	120,562 (45.9)	86,765 (45.7)	32,003 (46.1)	7827 (61.3)
Water	158,387	133,683	56,720	12,343
Average number of needles taken at each transaction	8.4	6.9	5.1	5.5
Estimated number of needles returned^a^ (% of total)	70,756 (27.0)	47,488 (25.0)	3198 (4.6)	16 (0.1)

^a^Recorded by IEP staff based on client estimates

#### Other injecting-related infections and injuries

Both the literature review and stakeholder feedback identified a link between public injecting and the risk of injecting-related infections and injury: for instance, due to disrupted hygiene routines, poor venous access in cold weather, and rushing to avoid detection [3, 6, 22, 27, 33, 34]. For instance, femoral vein (groin) injecting is often preferred for public injecting, as it is felt to be more rapid and reliable: however, it carries a greater risk of deep vein thrombosis, arterial puncture, and local or systemic infection.

“You’re outside, you’re freezing, you’re desperate, you’re in a hurry and you end up hitting an f***ing artery or something, do you know what I mean?”

*Focus group participant (in recovery).*

*“...you can get your groin easy, it’s in, out, two seconds…but if you’re trying to get a wee vein in your arms and you’re needing to get warm and get tourneys [tourniquets] on, you can’t do that kind of stuff in wee limited spaces or places you’ve got limited time.”*

*Interviewee (currently injecting drugs).*

#### Overdose and drug-related death

Our literature review identified an increased risk of overdose among people who inject drugs in public places [22, 23, 33, 35, 36], linked to rushed injecting to avoid detection or interruption [3, 25, 27]. Although current surveillance systems in the UK do not include data on injecting location, the demographic characteristics of people involved in public injecting in Glasgow (as shown in Table 2) match closely those most at risk of drug-related death [20].

### Existing service provision

Existing services for people who inject drugs in Glasgow span the four ‘tiers’ of addictions services described by the UK National Treatment Agency [37], including 9 multi-disciplinary Community Addictions Teams; specialist addictions teams for clients who are female, homeless or involved in the criminal justice system; addiction liaison teams in all acute hospitals; and 68 injecting equipment provision outlets. Local performance data suggest that the quality of service provision in Glasgow compares well to other areas of the UK and to international standards (Additional file 4).

### Potential novel interventions: safer injecting facilities

Our engagement exercise found that stakeholders were generally in favour of establishing a safer injecting facility in Glasgow, alluding to the evidence from other countries and identifying specific local benefits in the context of the HIV outbreak.

*“It’s a safe environment you’re in. You’re not in your close [stairwell], you’re not in a back alley where if anything happens there’s nobody there.”*

*Interviewee (currently injecting drugs).*

*“It has got to quite a ridiculous stage where members of the public, small businesses and communities are asking “why can’t you give these people somewhere safe to go and inject””*

*Senior staff, IEP services*

However, some stakeholders cited concerns about legality (since the operation of a SIF in the UK would contravene the Misuse of Drugs Act 1971 as it currently stands), public opinion, and operational risks.

*“If there are waiting times to access the facility there is no guarantee that the service user will use the facility consistently...”*

*Senior staff, Community Safety Glasgow*

*“[It] should not be seen as a panacea but rather part of a package of care to the most vulnerable population.”*

*Advocacy and support organisation leader*

### Potential novel interventions: heroin-assisted treatment

Analysis of secondary data (Table 2) and engagement responses suggested that a significant proportion of those involved in public injecting in Glasgow city centre may be eligible for – and benefit from – heroin-assisted treatment. In our consultation, stakeholders generally welcomed the prospect of HAT in Glasgow, citing the evidence for improved retention in addictions care and greater social stability, as well as a reduced risk from contaminated heroin. However, some raised concerns regarding cost and the potential for adverse public opinion.

*“I wish they would. Because you know something, it takes the smack out the city. It takes the illegal stuff out, and at least you know what you’re putting into your body.”*

*Interviewee (currently injecting drugs)*

*“You can put as many posters up as you like, saying that there is an increase in HIV in places. You need to think about it differently. That’s where I think safe injecting routes and injecting [prescribed] heroin…you take away the chaos. Then you have a chance to work on the attitude.”*

*Focus group participant (in recovery)*

*“Public perceptions of the programme may lead to greater stigma and possibly reduced engagement”*

*Manager, addictions services*

### Recommendations

Based on these findings, we made seven recommendations to address public injecting in Glasgow city centre:

1. Develop a strategy for cross-sectoral co-ordination between different agencies involved with this population, recognising the social determinants of public injecting.
2. Support the development of a peer network for harm reduction aimed at people in active drug use, building on successful local peer-led recovery initiatives.
3. Review models of delivery for specialist addiction services to ensure they can meet the complex needs of this population, with particular emphasis on access, engagement, and harm reduction.
4. Maximise out-of-hours access to injecting equipment through staffed outlets and peripatetic teams.
5. Introduce and evaluate a pilot safer injecting facility in the city centre, through close partnership working and integration with existing services.
6. Introduce and evaluate a pilot service for HAT for people who continue to use street heroin despite optimal opioid substitution therapy.
7. Incorporate questions on public injecting into routine assessments in existing services and national surveillance systems.

These recommendations have been endorsed by Glasgow City Alcohol and Drugs Partnership and work is currently underway to progress their implementation. Following an options appraisal, proposals are being developed by Glasgow City Health and Social Care Partnership to pilot the world’s first co-located safer injecting facility and heroin-assisted treatment service. This process will be described in more detail in a forthcoming study.

Challenges to implementation of the recommendations include: identification of a suitable location for the proposed facility; community engagement and consultation; and ensuring close integration with existing services. Furthermore, although HAT can be delivered legally within existing medicines legislation in the UK, the legal status of a safer consumption facility is more complex. At the time of writing, the Scottish Government has petitioned the UK Government to amend the Misuse of Drugs Act 1971, or to devolve drugs legislation to the Scottish Parliament, in order to permit the operation of such a facility: the UK Government has indicated it has no intention of doing so.

## Discussion

### Main findings of this study

This study found that public injecting in Glasgow city centre is prevalent and associated with significant health harms. Though the health needs identified in this assessment are universal among people who inject drugs, public injecting adds an additional layer of complexity and risk – both through the experience itself and through its close association with adverse social circumstances such as homelessness. Responses to public injecting must therefore address its ‘upstream’ determinants as well as mitigating the resultant harms. Among people with lived experience and staff from relevant health and social care services, there was widespread – though not unanimous – support for the local implementation of new harm reduction interventions such as safer injecting facilities and heroin-assisted treatment.

### What is already known?

Public injecting has been reported in a number of cities worldwide. Our findings corroborate previous research suggesting that the environment and context in which drug consumption occurs has profound implications for health, and that public injecting is often a marker of deep social exclusion [2, 26, 38]. Although evidence-based interventions exist, the extent of implementation varies internationally. For instance, at present no area in the UK provides safer injecting facilities or access to heroin-assisted treatment programmes, despite recommendations from the UK Advisory Council on the Misuse of Drugs, the British Medical Association and the Faculty of Public Health, and national clinical guidelines for the management of drug dependence [14, 39–41].

### What this study adds

This is the first comprehensive profile of the demographic characteristics and health needs of people who inject drugs in public places in the UK for more than two decades [22]. We have also described for the first time attitudes of professional stakeholders to the implementation of safer injecting facilities and heroin-assisted treatment, interventions which have a strong evidence base but remain politically sensitive. Our methods and findings may be useful for colleagues elsewhere who wish to understand the scale of public injecting, its associated health harms, and how to implement relevant interventions.

### Limitations

Place of use is not recorded by any existing routine data sources on drug-related harms in Scotland or the rest of the UK. We therefore relied on proxy indicators for public injecting, such as contact with an Assertive Outreach service set up to meet the needs of this population. Some of our data sources were designed for the purpose of service delivery rather than surveillance or research: we took a number of steps to maximise the validity of these data, such as excluding individuals with < 5 transactions per year from injecting equipment provision data, and triangulating findings across multiple sources.

## Conclusions

The environment and context in which drug consumption occurs is a key determinant of harm, and is inextricably linked to upstream social factors. Public injecting therefore requires a multifaceted public health response. Though evidence-based interventions to reduce public injecting and related harms exist, their implementation is variable. Following this study, Glasgow plans to establish the world’s first co-located safer injecting facility and heroin-assisted treatment service; progress on implementation and the results of evaluation will be reported in due course.

## Additional files

Additional file 1: Literature review search strategies and results. Describes the search strategies and number of results for the literature review element of the project. (DOCX 37 kb)

Additional file 2: Question schedules for engagement exercise. Provides the interview schedule, focus group topic guide, and content of online survey used in the stakeholder engagement element of the project. (DOCX 24 kb)

Additional file 3: Estimating the prevalence of public injecting. Estimating the prevalence of public injecting. (DOCX 13 kb)

Additional file 4: Scope and quality of existing service provision. Provides additional qualitative and quantitative data on the nature of existing service provision in Glasgow for the population of interest. (DOCX 17 kb)

## Abbreviations

HAT: Heroin-assisted treatment
HIV: Human Immunodeficiency Virus
IEP: Injecting equipment provision
SIF: Safer (or supervised) injecting facilities
