# Supplementary material for: “Taking away the chaos”: a health needs assessment for people who inject drugs in public places in Glasgow, Scotland
Source: BMC Public Health. 2018 Jul 4;18:829. doi: 10.1186/s12889-018-5718-9 (PMC6030790; doi:10.1186/s12889-018-5718-9)
Supplement: Supplementary file 1 — Literature review search strategies and results. Describes the search strategies and number of results for the literature review element of the project. (DOCX 37 kb) [file 12889_2018_5718_MOESM1_ESM.docx]

**ADDITIONAL FILE 1**

**Literature review search strategies and results**

Search 1: What is the estimated prevalence of public injecting among people who inject drugs in the UK and other high-income countries, and what are the health needs of this group?

**Medline**

|  | **Search terms** | **Results** |
| --- | --- | --- |
| 1 | exp Substance Abuse, Intravenous/ | 13319 |
| 2 | Inject$ drug use$.mp. | 8312 |
| 3 | (inject$ adj2 drug use$).mp. | 8396 |
| 4 | intravenous drug abuse.mp. | 980 |
| 5 | 1 or 2 or 3 or 4 | 17068 |
| 6 | (public adj2 place$).mp. | 1662 |
| 7 | (public adj2 space$).mp. | 462 |
| 8 | 6 or 7 | 2098 |
| 9 | 5 and 8 | 39 |
| 10 | open drug scene.mp. | 17 |
| 11 | (public adj2 inject$).mp. | 80 |
| 12 | 9 or 10 or 11 | 116 |

**Embase**

|  | **Search terms** | **Results** |
| --- | --- | --- |
| 1 | exp substance abuse/ | 44561 |
| 2 | exp intravenous drug administration/ | 360553 |
| 3 | exp intravenous drug abuse/ | 8868 |
| 4 | inject$ drug use$.mp. | 10569 |
| 5 | (inject adj2 drug use$).mp. | 24 |
| 6 | 1 or 2 or 3 or 4 or 5 | 419233 |
| 7 | (public adj2 place$).mp. | 2376 |
| 8 | (public adj2 space$).mp. | 658 |
| 9 | 7 or 8 | 2998 |
| 10 | 6 and 9 | 59 |
| 11 | open drug scene.mp. | 28 |
| 12 | (public adj2 inject$).mp. | 118 |
| 13 | 10 or 11 or 12 | 183 |

**Health Management Information Consortium**

|  | **Search terms** | **Results** |
| --- | --- | --- |
| 1 | exp Intravenous drugs/ | 127 |
| 2 | exp Drug abuse/ | 2437 |
| 3 | inject$ drug use$.mp. | 9 |
| 4 | drug consumption/ | 570 |
| 5 | 1 or 2 or 3 or 4 | 2971 |
| 6 | (public adj2 Place$).mp. [mp=title, other title, abstract, heading words] | 224 |
| 7 | 5 and 6 | 1 |
| 8 | open drug scene.mp. | 1 |
| 9 | (public adj2 Inject$).mp. [mp=title, other title, abstract, heading words] | 5 |
| 10 | 7 or 8 or 9 | 6 |

**Cinahl**

|  | **Search terms** | **Results** |
| --- | --- | --- |
| 1 | (MM "Substance Abuse, Intravenous") | 2352 |
| 2 | (MM "Intravenous Drug Users") | 858 |
| 3 | injecting drug user | 892 |
| 4 | 1 or 2 or 3 | 3372 |
| 5 | (MM "Public Spaces+") | 625 |
| 6 | public place$ | 558 |
| 7 | 5 or 6 | 1115 |
| 8 | 4 and 7 | 16 |
| 9 | Open drug scene | 9 |
| 10 | Public injecting | 24 |
| 11 | Injecting in public | 12 |
| 12 | 8 or 9 or 10 or 11 | 47 |

**Combined search results**

| **Total results of searches after de-duplication** | **182** |
| --- | --- |
| **Prevalence** |  |
| Total relevant results from searches | 16 |
| Additional relevant papers identified from reference lists | 2 |
| **Health needs** |  |
| Total relevant results from searches | 27 |
| Additional relevant papers identified from reference lists | 2 |

**Search 2:** What are the health impacts, social impacts and cost-effectiveness of safer injecting facilities?

**Medline**

| 1 | drug consumption room.mp. | 13 |
| --- | --- | --- |
| 2 | drug consumption facility.mp. | 4 |
| 3 | drug consumption facilities.mp. | 4 |
| 4 | safer injecting facility.mp. | 15 |
| 5 | safer injecting facilities.mp. | 9 |
| 6 | safer injection facility.mp. | 12 |
| 7 | safer injection facilities.mp. | 16 |
| 8 | supervised injection service.mp. | 1 |
| 9 | supervised injection services.mp. | 6 |
| 10 | supervised injecting centre.mp. | 17 |
| 11 | supervised injecting centres.mp. | 2 |
| 12 | supervised injecting service.mp. | 0 |
| 13 | supervised injecting services.mp. | 0 |
| 14 | supervised injection centre.mp. | 0 |
| 15 | supervised injection centres.mp. | 0 |
| 16 | Drug Consumption site.mp. | 0 |
| 17 | drug consumption sites.mp. | 0 |
| 18 | safer injecting site.mp. | 0 |
| 19 | safer injecting sites.mp. | 0 |
| 20 | safer injection site.mp. | 0 |
| 21 | safer injection sites.mp. | 1 |
| 22 | supervised injection site.mp. | 8 |
| 23 | supervised injection sites.mp. | 8 |
| 24 | supervised injecting site.mp. | 0 |
| 25 | supervised injecting sites.mp. | 1 |
| 26 | 1 or 2 or 3 or 4 or 5 or 6 or 7 or 8 or 9 or 10 or 11 or 12 or 13 or 14 or 15 or 16 or 17 or 18 or 19 or 20 or 21 or 22 or 23 or 24 or 25 | 96 |

**Embase**

| 1 | drug consumption room.mp. | 21 |
| --- | --- | --- |
| 2 | drug consumption facility.mp. | 5 |
| 3 | drug consumption facilities.mp. | 6 |
| 4 | safer injecting facility.mp. | 17 |
| 5 | safer injecting facilities.mp. | 11 |
| 6 | safer injection facility.mp. | 15 |
| 7 | safer injection facilities.mp. | 21 |
| 8 | supervised injection service.mp. | 7 |
| 9 | supervised injection services.mp. | 14 |
| 10 | supervised injecting centre.mp. | 25 |
| 11 | supervised injecting centres.mp. | 4 |
| 12 | supervised injecting service.mp. | 0 |
| 13 | supervised injecting services.mp. | 0 |
| 14 | supervised injection centre.mp. | 0 |
| 15 | supervised injection centres.mp. | 1 |
| 16 | Drug Consumption site.mp. | 0 |
| 17 | drug consumption sites.mp. | 0 |
| 18 | safer injecting site.mp. | 0 |
| 19 | safer injecting sites.mp. | 0 |
| 20 | safer injection site.mp. | 2 |
| 21 | safer injection sites.mp. | 2 |
| 22 | supervised injection site.mp. | 14 |
| 23 | supervised injection sites.mp. | 11 |
| 24 | supervised injecting site.mp. | 0 |
| 25 | supervised injecting sites.mp. | 1 |
| 26 | 1 or 2 or 3 or 4 or 5 or 6 or 7 or 8 or 9 or 10 or 11 or 12 or 13 or 14 or 15 or 16 or 17 or 18 or 19 or 20 or 21 or 22 or 23 or 24 or 25 | 138 |

**Health Management Information Consortium**

| 1 | drug consumption room.mp. | 1 |
| --- | --- | --- |
| 2 | drug consumption facility.mp. | 0 |
| 3 | drug consumption facilities.mp. | 0 |
| 4 | safer injecting facility.mp. | 2 |
| 5 | safer injecting facilities.mp. | 0 |
| 6 | safer injection facility.mp. | 2 |
| 7 | safer injection facilities.mp. | 1 |
| 8 | supervised injection service.mp. | 0 |
| 9 | supervised injection services.mp. | 0 |
| 10 | supervised injecting centre.mp. | 1 |
| 11 | supervised injecting centres.mp. | 2 |
| 12 | supervised injecting service.mp. | 0 |
| 13 | supervised injecting services.mp. | 0 |
| 14 | supervised injection centre.mp. | 0 |
| 15 | supervised injection centres.mp. | 0 |
| 16 | Drug Consumption site.mp. | 0 |
| 17 | drug consumption sites.mp. | 0 |
| 18 | safer injecting site.mp. | 0 |
| 19 | safer injecting sites.mp. | 0 |
| 20 | safer injection site.mp. | 0 |
| 21 | safer injection sites.mp. | 0 |
| 22 | supervised injection site.mp. | 0 |
| 23 | supervised injection sites.mp. | 0 |
| 24 | supervised injecting site.mp. | 0 |
| 25 | supervised injecting sites.mp. | 0 |
| 26 | 1 or 2 or 3 or 4 or 5 or 6 or 7 or 8 or 9 or 10 or 11 or 12 or 13 or 14 or 15 or 16 or 17 or 18 or 19 or 20 or 21 or 22 or 23 or 24 or 25 | 8 |

**Cinahl**

| 1 | "drug consumption room" | 9 |
| --- | --- | --- |
| 2 | "drug consumption facility" | 2 |
| 3 | "drug consumption facilities" | 3 |
| 4 | "drug consumption site" | 0 |
| 5 | "drug consumption sites" | 0 |
| 6 | "safer injecting facility" | 10 |
| 7 | "safer injecting facilities" | 6 |
| 8 | "safer injection facility" | 10 |
| 9 | "safer injection facilities" | 6 |
| 10 | "safer injecting sites" | 0 |
| 11 | "safer injecting sites" | 0 |
| 12 | "safer injection site" | 0 |
| 13 | "safer injection sites" | 2 |
| 14 | "supervised injection service" | 0 |
| 15 | "supervised injection services" | 4 |
| 16 | "supervised injecting service" | 0 |
| 17 | "supervised injecting services" | 0 |
| 18 | "supervised injection centre" | 0 |
| 19 | "supervised injection centres" | 0 |
| 20 | "supervised injecting centre" | 16 |
| 21 | "supervised injecting centres" | 3 |
| 22 | "supervised injecting site" | 0 |
| 23 | "supervised injecting sites" | 0 |
| 24 | "supervised injection sites" | 6 |
| 25 | "supervised injection site" | 2 |
| 26 | 1 OR 2 OR 3 OR 4 OR 5 OR 6 OR 7 OR 8 OR 9 OR 10 OR 11 OR 12 OR 13 OR 14 OR 15 OR 16 OR 17 OR 18 OR 19 OR 20 OR 21 OR 22 OR 23 OR 24 OR 25 | 71 |

**All databases combined**

| 1 | supervised injection facility.mp. [mp=ti, ab, ot, nm, hw, kf, px, rx, ui, an, tn, dm, mf, dv, kw] | 119 |
| --- | --- | --- |
| 2 | supervised injection facilities.mp. [mp=ti, ab, ot, nm, hw, kf, px, rx, ui, an, tn, dm, mf, dv, kw] | 57 |
| 3 | supervised injecting facility.mp. [mp=ti, ab, ot, nm, hw, kf, px, rx, ui, an, tn, dm, mf, dv, kw] | 42 |
| 4 | supervised injecting facilities.mp. [mp=ti, ab, ot, nm, hw, kf, px, rx, ui, an, tn, dm, mf, dv, kw] | 65 |
| 5 | 1 or 2 or 3 or 4 | 223 |
| 6 | remove duplicates from 5 | 134 |

**Total references retrieved after combination and de-duplication: 262**

**Search 3:** What are the health impacts, social impacts and cost-effectiveness of providing heroin-assisted treatment?

**Medline**

|  | **Search terms** | **Results** |
| --- | --- | --- |
| 1 | heroin assisted treatment.mp. | 67 |
| 2 | HAT.mp. | 5392 |
| 3 | exp Heroin Dependence/ or exp Heroin/ | 11832 |
| 4 | 2 and 3 | 23 |
| 5 | 1 or 4 | 68 |
| 6 | prescribed heroin.mp. [mp=title, abstract, original title, name of substance word, subject heading word, keyword heading word, protocol supplementary concept word, rare disease supplementary concept word, unique identifier] | 19 |
| 7 | Heroin/ad [Administration & Dosage] | 1044 |
| 8 | Prescription Drugs/ad [Administration & Dosage] | 452 |
| 9 | Narcotics/ad [Administration & Dosage] | 2528 |
| 10 | Methadone/ad [Administration & Dosage] | 2067 |
| 11 | 7 and 8 and 9 and 10 | 2 |
| 12 | 5 or 6 or 11 | 87 |
| 13 | supervised injectable heroin treatment.mp. | 1 |
| 14 | 12 or 13 | 88 |

**Embase**

|  | **Search terms** | **Results** |
| --- | --- | --- |
| 1 | heroin assisted treatment.mp. | 106 |
| 2 | HAT.mp. | 8447 |
| 3 | diamorphine/ | 20296 |
| 4 | 2 and 3 | 34 |
| 5 | 1 or 4 | 109 |
| 6 | prescribed heroin.mp. | 27 |
| 7 | diamorphine/ad [Drug Administration] | 491 |
| 8 | prescription drug/ad [Drug Administration] | 156 |
| 9 | 7 and 8 | 2 |
| 10 | supervised injectable heroin treatment.mp. | 2 |
| 11 | 5 or 6 or 9 or 10 | 137 |

**Health Management Information Consortium**

|  | **Search terms** | **Results** |
| --- | --- | --- |
| 1 | Heroin assisted treatment.mp. | 3 |
| 2 | HAT.mp. | 41 |
| 3 | exp Heroin/ | 216 |
| 4 | 2 and 3 | 2 |
| 5 | 1 or 4 | 3 |
| 6 | prescribed heroin.mp. | 1 |
| 7 | exp Heroin/ | 216 |
| 8 | exp Narcotics/ | 360 |
| 9 | exp Methadone/ | 120 |
| 10 | 7 or 8 or 9 | 430 |
| 11 | exp Drug administration/ | 2189 |
| 12 | 10 and 11 | 16 |
| 13 | 5 or 6 or 12 | 20 |

**Cinahl**

|  | **Search terms** | **Results** |
| --- | --- | --- |
| 1 | "heroin assisted treatment" | 35 |
| 2 | "HAT" | 458 |
| 3 | (MH "Heroin") | 1918 |
| 4 | 2 AND 3 | 6 |
| 5 | "prescribed heroin" | 8 |
| 6 | (MH "Heroin/AD") | 258 |
| 7 | (MH "Methadone/AD") | 959 |
| 8 | 6 AND 7 | 25 |
| 9 | "supervised injectable heroin treatment" | 2 |
| 10 | 1 OR 4 | 35 |
| 11 | 5 OR 8 OR 9 OR 10 | 66 |

**Total references retrieved after combination and de-duplication: 271**

**Search 4:** What are the health impacts, social impacts and cost-effectiveness of expanding access to injecting equipment provision?

**Medline**

|  | **Search terms** | **Results** |
| --- | --- | --- |
| 1 | Needle-Exchange Programs/mt [Methods] | 44 |
| 2 | Needle-Exchange Programs/sd [Supply & Distribution] | 34 |
| 3 | Needle-Exchange Programs/og [Organization & Administration] | 211 |
| 4 | Syringes/ | 5371 |
| 5 | vending machine.mp. | 113 |
| 6 | 4 and 5 | 2 |
| 7 | dispensing machine.mp. | 21 |
| 8 | 4 and 7 | 1 |
| 9 | syringe program$.mp. | 167 |
| 10 | exp Substance-Related Disorders/ | 237097 |
| 11 | 9 and 10 | 152 |
| 12 | access to sterile syringes.mp. | 66 |
| 13 | inject$ equipment provision.mp. | 5 |
| 14 | 10 and 12 | 62 |
| 15 | 24 hours.mp. | 80208 |
| 16 | out of hours.mp. | 1282 |
| 17 | extended hours.mp. | 93 |
| 18 | 15 or 16 or 17 | 81513 |
| 19 | 4 and 10 and 18 | 2 |
| 20 | 1 or 2 or 3 or 6 or 8 or 11 or 13 or 14 or 19 | 466 |
| 21 | Access to Sterile Needle$.mp. | 12 |
| 22 | 10 and 21 | 11 |
| 23 | 20 or 22 | 476 |

**Embase**

|  | **Search terms** | **Results** |
| --- | --- | --- |
| 1 | needle exchange program$.mp. | 614 |
| 2 | syringe exchange program$.mp. | 439 |
| 3 | syringe/ | 11505 |
| 4 | vending machine.mp. | 170 |
| 5 | 3 and 4 | 5 |
| 6 | dispensing machine.mp. | 42 |
| 7 | 3 and 6 | 4 |
| 8 | 1 or 2 | 1018 |
| 9 | exp substance abuse/ | 44672 |
| 10 | 8 and 9 | 212 |
| 11 | access to sterile syringe$.mp. | 81 |
| 12 | 9 and 11 | 24 |
| 13 | Inject$ Equipment Provision.mp. | 7 |
| 14 | access to sterile needle$.mp. | 14 |
| 15 | 24 hour.mp. | 51350 |
| 16 | out of hours.mp. | 2370 |
| 17 | extended hours.mp. | 147 |
| 18 | 15 or 16 or 17 | 53801 |
| 19 | 3 and 9 and 18 | 0 |
| 20 | 5 or 7 or 10 or 12 or 13 or 14 | 250 |

**Health Management Information Consortium**

|  | **Search terms** | **Results** |
| --- | --- | --- |
| 1 | exp Needle exchange schemes/ | 45 |
| 2 | exp Needles for injection/ | 137 |
| 3 | exp Syringes/ | 169 |
| 4 | 2 or 3 | 277 |
| 5 | exp Vending machines/ | 30 |
| 6 | 4 and 5 | 0 |
| 7 | dispensing machine.mp. | 1 |
| 8 | 4 and 7 | 0 |
| 9 | syringe program$.mp. | 13 |
| 10 | access to sterile syringes.mp. | 2 |
| 11 | inject$ equipment provision.mp. | 0 |
| 12 | access to sterile needles.mp. | 0 |
| 13 | 1 or 9 or 10* | 57 |
| 14 | exp "Out of hours health services"/ | 861 |
| 15 | 24 hours.mp. | 454 |
| 16 | extended hours.mp. | 37 |
| 17 | 14 or 15 or 16 | 1332 |
| 18 | 4 and 17 | 0 |

***Used as final results**

**Cinahl**

|  | **Search terms** | **Results** |
| --- | --- | --- |
| 1 | (MH "Needle Exchange Programs/AM/EV/MT") | 116 |
| 2 | (MH "Syringes") | 1682 |
| 3 | (MH "Needles") | 3218 |
| 4 | S2 OR S3 | 4649 |
| 5 | "vending machine" | 70 |
| 6 | "dispensing machine" | 8 |
| 7 | S5 OR S6 | 78 |
| 8 | S4 AND S7 | 0 |
| 9 | "syringe program" | 29 |
| 10 | "syringe programs" | 51 |
| 11 | S9 OR S10 | 74 |
| 12 | "access to sterile syringes" | 21 |
| 13 | "access to sterile needles" | 5 |
| 14 | "injection equipment provision" | 0 |
| 15 | "24 hours" | 10277 |
| 16 | "out of hours" | 657 |
| 17 | "extended hours" | 57 |
| 18 | 15 OR 16 OR 17 | 10984 |
| 19 | 4 AND 18 | 14 |
| 20 | 1 OR 11 OR 12 OR 13 OR 19 | 221 |

AM = Administration EV = Evaluation MT = Methods

**Total references retrieved after combination and de-duplication: 858**
