# Supplementary material for: “Taking away the chaos”: a health needs assessment for people who inject drugs in public places in Glasgow, Scotland
Source: BMC Public Health. 2018 Jul 4;18:829. doi: 10.1186/s12889-018-5718-9 (PMC6030790; doi:10.1186/s12889-018-5718-9)
Supplement: Supplementary file 2 — Question schedules for engagement exercise. Provides the interview schedule, focus group topic guide, and content of online survey used in the stakeholder engagement element of the project. (DOCX 24 kb) [file 12889_2018_5718_MOESM2_ESM.docx]

# ADDITIONAL FILE 2. Question schedules for engagement exercise.

**A2.1. Interview schedule for people currently involved in injecting drug use**

| **MAIN QUESTIONS** | | **PROMPTS & EXTRA QUESTIONS** |
| --- | --- | --- |
| **INTRODUCTION** | | |
| *Interviewer introduces themselves and their role.*  The NHS and Glasgow City Council are currently reviewing the services they provide for people who inject drugs in the city centre so are carrying out interviews to find about people’s views and experiences.  By taking part in this interview, you will be helping us improve the health services that we provide. In particular, we are interested in issues around where injection takes place and the risks that people experience when injecting in public.  The interview will last about an hour, and you will be provided with a voucher as compensation for your time.  There are no right or wrong answers: we are interested in your opinions and your experiences.  Taking part is voluntary: you can decline to answer any question or to stop the interview at any time without giving a reason.  We will be recording the interview so that we can recall your views accurately: this recording will only be heard by public health staff working on the project – not by any staff from addictions services - and will be deleted after use.  You will not be identified by name in any of the work that comes out of these interviews and none of the information you provide will be shared with anyone involved in your health care or social care.  Are you happy to take part? | | |
| **BREAKING THE ICE** | | |
| **Perhaps you could start by telling me a bit about yourself.**  **What’s your current housing situation?**  Are you in work right now?  What benefits do you receive?  **Could you tell me about your drug use?** | | How old are you? Where are you from?  For drug use:  What? How long for?  How often? How much?  Where? |
| **INJECTING BEHAVIOUR** | | |
| If living outside city centre:   - How often do you come into the city centre? - How far is it from where you live?   **On average, how many times per day do you use drugs in the city centre?**  On average, how many people do you prepare and inject with?  **What locations do you use to inject? Why do you use these?**  **Does preparing and injecting your drugs away from home change the process at all?** | e.g. Car parks? Toilets? Waste ground? Alleyways?  e.g. re-use of needles? Hand-washing? Access to water? | |
| **NEEDS** | | |
| **How would you describe your general health?**  How high on your list of priorities is health?  Do any health issues worry you right now?  Is there any kind of help you would like to improve your health?  **Are there any barriers that stop you from having better health?** | Over the past year, have you had any of the following?   - Overdose - Abscess - Deep vein thrombosis (blood clot) - Ulcer or slow-healing wounds - Diagnosis of blood borne virus e.g. hep C, HIV | |
| **EXISTING SERVICES** | | |
| **What kinds of help have you received for your drug use?**  **How did you find it?**  **If you could change anything about these services, what would you change?** | e.g. methadone, needle exchange, detox, rehab, counselling   - Where and from whom? - When? - For how long? - What were the positive things about these services? - What were the negative things about these services? | |
| If user reports not having used any relevant services;   - Is there any reason that you haven’t been involved with any services?   How would you want these services to change to better meet your needs? | | |
| **OTHER SERVICES** | | |
| Are there any services that are not currently available in Glasgow but that you would like to see here?  **Some other countries provide safe injecting rooms. These are clean and hygienic indoor facilities supervised by a nurse or doctor where people can inject drugs they have bought elsewhere. They aim to reduce the risk of infections and overdoses and to provide a place where people can access other services like advice on housing or benefits.**   - **What do you think about the idea of introducing a service like this in Glasgow?** - Is it a service you would be interested in using? Why/why not? - Would an option for something like this, a place where you could inject safely, reduce the chances of you injecting in public? - What impact do you think this would have on your health? - [*If positive to idea*] What would that facility look like in terms of opening hours, location, services offered etc?   **Some other countries provide heroin assisted treatment. This is when doctors prescribe injectable heroin to people for whom methadone or suboxone treatment hasn’t worked.**   - **What do you think about the idea of introducing a service like this in Glasgow?** - Is this a treatment you would consider? Why/why not? - What impact do you think this would have on your health? - [*If positive to the idea*] How would it be beneficial to you over other existing treatments?   **Another option would be making sure that people could get hold of clean injecting equipment in the city centre in the evenings and overnight, for example via a vending machine or a 24h pharmacy in the city centre.**   - **Is this something you would like to see in Glasgow? Why/why not?** - Would you use this service? - What impact do you think this would have on your health? - [*If positive to the idea*] How would it be beneficial to you over other needle exchange services? | | |
| **CONCLUSION** | | |
| Is there anything else you’d like to tell me?  *Thank interviewee for taking part & provide voucher.* | | |

**A2.2. Focus group topic guide for people in recovery from injecting drug use**

| **MAIN QUESTIONS** | **PROMPTS** |
| --- | --- |
| **Introduction** |  |
| Hello, our names are [**NAMES**] and we are [**ROLES**].  Thanks for coming along to this focus group today.  The aim of today is to explore health issues associated with injecting drugs. In particular, we’ll be talking about what people need in terms of health care, the services that exist at present, and potential new services that aren’t currently provided.  We want to hear your views: there’s no right or wrong answer. We want you to be able to say exactly what you think. We would therefore ask you to be respectful to everyone else in the group and what they have to say.  I have a list of questions we’ll be discussing but free to respond to questions and to others points without being called upon. However, I would ask that only one person speak at a time. There will be a lot to talk about, so at times I may move the discussion along a bit.  We’ll be writing things up on the flipchart but if you have anything you’d like to contribute but would rather not share with the group, either write it on a post-it or speak to us at the break or the end.  We’re scheduled to meet until 4pm but will have a comfort break halfway through. Toilets are located…Please help yourself to refreshments as you need them.  We are recording this discussion so we don’t miss anything you have to say, but you will not be identified in the recording or in any reports we produce later on, so no-one else will know who said what, and the tape will be destroyed later on. If you wish to leave at any point, you can of course do so, without having to give a reason.  Does anyone have any questions?  Is everyone happy to continue? |  |
| **Breaking the ice** |  |
| **Perhaps we could start by each spending a couple of minutes telling us a little bit about yourself and your history.** | How old are you? Where are you from?   - When did you start using drugs? - What were your drugs of choice? - Where would you inject – home, friend’s houses, outdoors? - What help did you receive? |
| **Needs** |  |
| **I now want to ask you to think back to when you were using and think about what your health was like, and how it could have been improved.**  **So, thinking back to when you were using:**   - How high on your list of priorities was health? - **What were the main health issues you experienced?** - Can you think of anything that stopped you having better health? |  |
| **Break** |  |
| **Existing services** |  |
| **Thinking back to when you were using…**  **What was your experience of addictions services?**  What were the positive things about that service?  What were the negative things about that service?  **If you could change anything about that service, what would you change?** | Ensure specifically discuss:   - Substitution therapy (methadone) - IEP - Community addiction team support |
| If users report not having accessed any relevant services;   - Is there any reason that you didn’t access those services? - How could those services have better met your needs when you were using? |  |
| **Other services** |  |
| **Are there any services for people who inject drugs that aren’t currently available in Glasgow but you would like to see?**  **Some other countries provide facilities called ‘safer injecting sites’ or ‘drug consumption rooms’. These are clean and hygienic indoor facilities supervised by a nurse or doctor where people can inject drugs they have bought elsewhere. They aim to reduce the risk of infections and overdoses and to provide a place where people can access other services like advice on housing or benefits.**  **What are your thoughts on this?**  **Some other countries provide heroin on prescription. This is when doctors prescribe injectable heroin to people for whom methadone or suboxone treatment hasn’t worked.**  **What are your thoughts on this?**  **At present in Glasgow city centre, injecting equipment is available from pharmacies during the day, or the crisis centre overnight and at weekends. It has been suggested that injecting equipment should be made more readily accessible, for example by 24 hour services in the city centre or through the use of vending machines.**  **What are your thoughts on this?** | Can you describe how that would have helped you stay healthier?  Would this have been a service you would have accessed when you were using?  [If in favour] What should a service like this look like in terms of location, opening hours, facilities etc?  Do you think this would reduce the likelihood of people injecting in public? Why/why not?  **What do you think might be the benefits or risks of having a service like this in Glasgow?**  Would this have been a treatment you would have been willing to consider when you were using?  **What do you think might be the benefits or risks of having a service like this in Glasgow?**  Would this have been a service you would have accessed when you were using?  **What do you think might be the benefits or risks of having a service like this in Glasgow?**  Do you think this would reduce the likelihood of people sharing tools? Why/why not? |
| **Conclusion** |  |
| Is there anything else we haven’t discussed that anyone would like to mention?  Thank you all very much for taking part. It has been very useful to hear your views, and we’re very grateful for your time and contribution.  If you would like to be kept informed of the results of this project, please write down your contact details on this sheet and we will send you a summary once the project is complete. |  |

**A2.3. Online engagement exercise for staff of relevant health and community services**

Personal Information

1. Name (optional):

The following questions are about your role.

1. Job role:

- Addictions case worker
- Addictions nurse
- Addictions physician
- Addictions service manager
- Advocacy or support organisation leader
- Homeless practice GP
- Homeless practice nurse
- Homeless practice manager
- Infectious disease physician
- Injecting equipment provision - service manager
- Injecting equipment provision - pharmacist
- Outreach worker
- Recovery group co-ordinator
- Sexual health advisor
- Other (please specify)

1. Organisation:
2. Time in Post:

- Less than 6 months
- 6 months to 1 year
- 1 year to 3 years
- 3 years to 6 years
- 6 years to 10 years
- More than 10 years

1. Brief description of the role and associated responsibilities:
2. How often do you work with people who inject drugs?

- Every day
- Most days
- Every few weeks
- Every few months
- A few times per year
- Never

The following questions relate to people who inject drugs in public places in Glasgow city centre.

1. Please list what you think are the **three** most important health concerns of this group.
2. Please list what you think are the **three** most important unmet needs of this group in relation to health.

The following questions relate to **existing services.**

1. How well do you think your service meets the needs of this population at present?

- Very well
- Well
- Satisfactorily
- Poorly
- Very poorly

1. What are the positive aspects of the current set-up?
2. What are the negative aspects of the current set-up?
3. What barriers exist for this population in accessing your service at present?

(such as opening hours, location, appointment systems, etc)

1. What could be done to overcome these?

The following questions relate to the introduction of novel services for people who inject drugs in public places in Glasgow city centre.

**Safer Injecting Facilities**

‘Safer injecting facilities’ - also known as drug consumption rooms or medically supervised injecting sites – are clinically supervised areas that provide a hygienic environment where dependent individuals can consume drugs, in order to reduce the individual and social harms of public injecting.

More information on safer injecting facilities can be found at:

<http://www.emcdda.europa.eu/topics/pods/drug-consumption-rooms>

1. What is your attitude towards the potential introduction of Safer Injecting Facilities in Glasgow city centre?

- Very positive
- Positive
- Neutral
- Negative
- Very negative

1. In particular, what do you think would be the potential benefits of this intervention?
2. In particular, what do you think would be the potential harms or risks of this intervention?
3. What impact do you think this intervention would have on public injecting in Glasgow?
4. What impact do you think this intervention would have on the wider community in Glasgow?
5. Any other comments on Safer Injecting Facilities?

**Heroin-assisted treatment**

Heroin-assisted treatment refers to the prescription of injectable medical-grade heroin to people with opiate dependency who have not benefited from other opiate replacement therapies such as methadone or buprenorphine. More information on heroin-assisted treatment can be found at:

<http://www.emcdda.europa.eu/attachements.cfm/att_154996_EN_Heroin%20Insight.pdf>

1. What is your attitude towards the potential introduction of Heroin-Assisted Treatment in Glasgow city centre?

• Very positive

• Positive

• Neutral

• Negative

• Very negative

1. In particular, what do you think would be the potential benefits of this intervention?
2. In particular, what do you think would be the potential harms or risks of this intervention?
3. What impact do you think this intervention would have on public injecting in Glasgow?
4. What impact do you think this intervention would have on the wider community in Glasgow?
5. Any other comments on Heroin-Assisted Treatment?

**Expanding access to injecting equipment provision services**

1. What is your attitude towards expanding access to injecting equipment provision services in Glasgow city centre, for example through vending machines or increased out-of-hours provision?

• Very positive

• Positive

• Neutral

• Negative

• Very negative

1. In particular, what do you think would be the potential benefits of this intervention?
2. In particular, what do you think would be the potential harms or risks of this intervention?
3. What impact do you think this intervention would have on public injecting in Glasgow?
4. What impact do you think this intervention would have on the wider community in

Glasgow?

1. Any other comments on expanding access to injecting equipment provision?

**Concluding remarks**

1. Do you have any final comments to make on the issue of public injecting or the needs assessment that have not been covered in previous sections?

Thank you very much for taking part in this consultation.

Further information on the outcome of the needs assessment will be disseminated in due course.
