# Supplementary material for: “Taking away the chaos”: a health needs assessment for people who inject drugs in public places in Glasgow, Scotland
Source: BMC Public Health. 2018 Jul 4;18:829. doi: 10.1186/s12889-018-5718-9 (PMC6030790; doi:10.1186/s12889-018-5718-9)
Supplement: Supplementary file 3 — Estimating the prevalence of public injecting. Estimating the prevalence of public injecting. (DOCX 13 kb) [file 12889_2018_5718_MOESM3_ESM.docx]

**Additional file 3. Estimating the prevalence of public injecting.**

Published estimates of the prevalence of public injecting vary widely, even among the five studies carried out in the UK. Only one prevalence study including participants from Glasgow was identified [1]. In that study, of 398 individuals recruited from injecting equipment provision (IEP) services in Glasgow, London, and Leeds during 2005, 42% reported having injected at least once in public places during the last week.

During 2015, a total of 3,320 people who reported injecting heroin and/or cocaine accessed injecting equipment from the seven IEP outlets located in Glasgow city centre and neighbouring areas, including the Assertive Outreach team.

As described in the main paper, IEP data has not yet been validated for the purposes of estimating the population of people who inject drugs, and may be artificially inflated by individuals using more than one identifier. However, these were the most comprehensive data for the purpose of estimating the population of people who inject drugs available at the time of the needs assessment. If limited to clients with at least five transactions in city centre pharmacies during 2015 (‘repeat clients’), the figure falls to 1,025 (30.9% of total), suggesting a smaller population of unique clients using these outlets on a regular basis.

Applying prevalence estimates of people injecting drugs in public places from Hunt’s study of three UK cities to 2015 data from the seven IEP outlets in the city centre and surrounds yields an estimate of 1,394 people injecting in public places in this area on a weekly basis. However, when the IEP data is restricted to only ‘repeat clients’ (i.e. those with five or more transactions during 2015), the figure of people regularly injecting in public places falls to 431.

This number is consistent with records from the Assertive Outreach service, which made contact with 470 unique individuals between June 2014 and January 2016. It also concurs with the impressions of service providers in contact with this population.

However, this estimate has a number of limitations. Since it refers only to the past week, it may under-estimate public injecting, particularly among less frequent injectors. It is based on data from London and Leeds, as well as Glasgow: the extent to which the prevalence of public injecting varies across the UK is unknown. It is also unclear whether the prevalence of public injecting has changed in the decade since this research was undertaken. Given these limitations, the needs assessment recommended a number of measures to improve the availability and quality of data on public injecting, using existing surveillance systems (such as the Needle Exchange Surveillance Initiative) and routine assessments in addictions services.

1. Hunt N. Indicators of the need for drug consumption rooms in the UK (paper A). York: Joseph Rowntree Foundation; 2006.
