# Supplementary material for: “Taking away the chaos”: a health needs assessment for people who inject drugs in public places in Glasgow, Scotland
Source: BMC Public Health. 2018 Jul 4;18:829. doi: 10.1186/s12889-018-5718-9 (PMC6030790; doi:10.1186/s12889-018-5718-9)
Supplement: Supplementary file 4 — Scope and quality of existing service provision. Provides additional qualitative and quantitative data on the nature of existing service provision in Glasgow for the population of interest. (DOCX 17 kb) [file 12889_2018_5718_MOESM4_ESM.docx]

**Additional file 4. Scope and quality of existing service provision in Glasgow**

Scope of existing provision

The European Monitoring Centre on Drugs and Drug Addiction (EMCDDA) has identified a set of interventions which constitute evidence-based best practice for people who inject drugs [1, 2]. Most – but not all – of these are currently provided within the area covered by Glasgow City Alcohol and Drugs Partnership (ADP), including: opioid substitution therapy combined with psychosocial support; tapered opioid substitution therapy for detoxification; outreach-based harm reduction services; take-home naloxone for preventing overdose-related deaths; and low-threshold provision of sterile needles, syringes, and injecting paraphernalia.

Three EMCDDA-recommended interventions are not currently provided in Glasgow: safer injecting facilities (also known as drug consumption rooms); heroin-assisted treatment; or a comprehensive programme of peer-based harm reduction interventions.

Quality of existing provision

The Needle Exchange Surveillance Initiative (NESI) study has found that, among people attending injecting equipment provision (IEP) outlets in NHS Greater Glasgow and Clyde, the proportion of recent initiates to injecting drug use (onset of injecting within last 5 years) has declined, from 26% in 2008 to 17% in 2013 [REF]^[[1]](#footnote-1)^. The proportion of people injecting on a daily basis has also declined, from 61% in 2008 to 42% in 2013. This suggests that the incidence of injecting drug use – and the intensity of injecting – is declining over time locally.

National waiting time standards in Scotland mandate that at least 90% of people referred to alcohol and drug treatment services should wait no longer than three weeks before the start of appropriate treatment. In 2015, 96% of people referred to services in Glasgow City ADP started treatment for drug problems within three weeks of referral; this compares to a national average of 93.9% [REF].

Local addictions services work to service standards and prescribing guidelines derived from the UK guidelines for the clinical management of drug misuse and dependence [REF]. Estimates suggest that coverage of opioid substitution therapy (OST) in the Glasgow City ADP area – calculated by dividing the number of individuals prescribed OST by the estimated number of problem drug users resident in the area – is approximately 52% [REF]. This is comparable to the UK and European average [7], and above the World Health Organisation threshold for ‘high’ coverage of OST, defined as >40% [1,2,8]. However, it remains lower than the highest performing countries, such as Austria, Luxembourg, and Switzerland [8,9].

As highlighted above, the crude rate of drug-related deaths in Glasgow – expressed as deaths per 1,000 population – is among the highest in Scotland. However, with respect to rates of drug-related death per 1,000 problem drug users – a metric which takes into account the underlying prevalence of problem drug use and therefore better reflects the performance of health services in harm reduction – Glasgow is below the national average (8.1 per 1,000 vs 9.4 per 1,000) [10].

Local injecting equipment provision (IEP) services fulfil the EMCDDA standard – and World Health Organisation recommendations – for low-threshold provision of sterile needles, syringes, and injecting paraphernalia, with a wide range of equipment available on an unlimited basis from a large number of outlets in diverse settings [1,8].

Estimating the coverage of IEP services is methodologically challenging; this is particularly the case in Scotland, where estimates of the number of people who inject drugs have not been produced since 2006. Attempts to estimate local coverage – even using pragmatic definitions such as ‘service utilisation’, the number of needles distributed per injector per year [8] – produce very different results, depending on which estimate of the denominator population is used. However, NESI data from NHSGGC as a whole suggest that injecting risk behaviours – such as sharing of needles, syringes and injecting equipment – are declining over time [3]. For instance, the proportion of respondents reporting having injected with a needle or syringe previously used by someone else declined from 8% in 2008 to 3% in 2013. In keeping with this trend, hepatitis C antibody prevalence among recent initiates to injecting drug use (a marker of ongoing transmission) has also declined over time, from 32% in 2008 to 21% in 2013 [3].

**References**

1. European Monitoring Centre for Drugs and Drug Addiction. Best practice: harm reduction for opioid injectors. <http://www.emcdda.europa.eu/best-practice/answer-sheet/harm-reduction-opioid-injectors_en>. Accessed 6^th^ September 2017.
2. European Monitoring Centre for Drugs and Drug Addiction. Best practice: treatment for opioid users. <http://www.emcdda.europa.eu/best-practice/answer-sheets/treatment-opioid-users_en>. Accessed 6^th^ September 2017.
3. Munro A. Personal communication: NESI data for NHS GG&C, 2008-2013.
4. Information Services Division Scotland. National drug and alcohol treatment waiting times. Edinburgh: Information Services Division Scotland; 2016
5. Clinical Guidelines on Drug Misuse and Dependence Update 2017 Independent Expert Working Group (2017) Drug misuse and dependence: UK guidelines on clinical management. London: Department of Health.
6. Duncan S. Personal communication: Glasgow Addictions Service activity data, 2014-2015.
7. European Monitoring Centre for Drugs and Drug Addiction. European drug report: Trends and developments. Lisbon: EMCDDA; 2015.
8. WHO, UNODC, UNAIDS. Technical guide for countries to set targets for universal access to HIV prevention, treatmentand care for injecting drug users – 2012 revision. Geneva: World Health Organisation; 2012.
9. Besson J, Beck T, Wiesbeck G, Hammig R, Kuntz A, Abid S, Stohler R. Opioid maintenance therapy in Switzerland: An overview of the Swiss IMPROVE study. Swiss Medical Weekly 2014 Mar 31;144:w13933.
10. National Records of Scotland. Drug-related deaths in Scotland in 2015. Edinburgh: National Records for Scotland; 2016.

1. Note that NESI data on these indicators are unfortunately not available for the smaller geographical area covered by the Glasgow City Alcohol and Drugs Partnership. [↑](#footnote-ref-1)
